# circDENND4C serves as a sponge for miR-200b to drive non-small cell lung cancer advancement by regulating MMP-9 expression

**DOI:** 10.3389/fonc.2025.1441384

**Published:** 2025-02-17

**Authors:** Yaming Lv, Lan Wang, Yunhui Zhang, Dong Wei, Yajie Hu

**Affiliations:** ^1^ Department of Respiratory Medicine, The Affiliated Hospital of Kunming University of Science and Technology, Kunming, China; ^2^ Department of Respiratory Medicine, The First People’s Hospital of Yunnan Province, Kunming, China; ^3^ Department of Hepatopancreatobiliary Surgery, The Second Affiliated Hospital of Kunming Medical University, Kunming, China

**Keywords:** circDENND4C, miR-200b, MMP-9, non-small cell lung cancer (NSCLC), tumor progression

## Abstract

**Introduction:**

Lung cancer has a higher incidence and mortality rate than other cancers, especially non-small cell lung cancer (NSCLC), accounting for 85% of the cases. The role of the circDENND4C/miR-200b/matrix metalloproteinase-9 (MMP-9) regulatory axis in NSCLC remains largely unknown.

**Methods:**

NSCLC cell lines were used to examine the expression of circDENND4C, miR-200b, and MMP-9 via qRT-PCR or Western blot. The target relationship of circDENND4C, miR-200b, and MMP-9 was examined by RNA fluorescence *in situ* hybridization (RNA-FISH), immunofluorescence (IF), dual-luciferase reporter system, quantitative real-time polymerase chain reaction (qRT-PCR), and Western blot. Then, a cell count kit-8 (CCK-8) experiment, flow cytometry, and migration/invasion assays were performed to assess the biological function of circDENND4C, miR-200b, and MMP-9 by transfecting with their overexpression or knockout plasmids in A549 cells. Finally, the proteins related to cell adhesion and tight junction were further tested by Western blot and IF.

**Results:**

circDENND4C and MMP-9 were found to be highly expressed in NSCLC cell lines, while miR-200b was lowly expressed in NSCLC cell lines. Moreover, circDENND4C could sponge miR-200b to target MMP-9. Subsequently, it was observed that knockdown of circDENND4C and MMP-9 or the upregulation of miR-200b repressed cell proliferation and cell cycle progression, increased cell apoptosis, and hindered cell migration and invasion. Finally, it was also found that the circDENND4C/miR-200b/MMP-9 regulatory axis might be involved with cell adhesion and tight junction to influence tumor metastasis.

**Conclusions:**

Altogether, our study reveals a novel regulatory loop in which the circDENND4C/miR-200b/MMP-9 axis may modulate NSCLC progression, indicating potential biomarkers for the diagnosis or treatment of NSCLC.

## 1 Introduction

Lung cancer has become one of the most common dangerous tumors and is also an important factor for tumor-associated death around the world. Non-small cell lung cancer (NSCLC) and small cell lung cancer (SCLC) are two common subtypes of lung cancer (1, 2). Overall, 80%–85% of all human lung cancers are NSCLC, which mainly contains three major sub-types: squamous cell lung carcinoma, lung adenocarcinoma, and large cell lung carcinoma, and is the leading cause of lung cancer-associated mortality (3, 4). In China, the incidence and mortality of lung cancer have constantly increased each year (5, 6). Because of insufficient screening and the lack of early clinical symptoms, clinicopathological features, or a relatively specific tumor marker for the diagnosis, most patients with NSCLC are diagnosed at late stages and are put in a very disadvantageous situation. Moreover, patients with lung cancer have a very high rate of recurrence and metastasis, which leads to a very poor prognosis (7, 8). In the past decades, despite the advancement in the diagnosis of imaging techniques, sputum cytology analysis, and targeted drugs for specific gene mutations of lung cancer, the 5-year overall survival rate of patients with lung cancer is lower than 20% (9, 10). Therefore, it is of great significance to explore the molecular mechanisms of lung cancer, which not only reveal novel biomarkers for an effective early screening of lung cancer or determine new targets for the treatment of lung cancer, but also help us further understand the progression of lung cancer to develop a new management model.

Current studies have reported that with the advancement of high-throughput sequencing technology and bioinformatics methods, there were a large number of non-coding RNAs (ncRNAs) identified. ncRNAs, including microRNA (miRNA), long non-coding RNA (lncRNA), and circular RNA (circRNA), are transcribed from more than 90% of human transcripts and regulate gene expression (11, 12). circRNA, a class of single-stranded, endogenous and conserve ncRNA, forms a covalently closed continuous loop via back-splice without 3’-end or 5’-end (13) and plays an essential role to regulate gene expression at the post-transcriptional level (14). circRNAs act as competing endogenous RNA (ceRNA) by sponging miRNA to relieve the repression of miRNAs for their targets (14, 15). Recently, circRNAs have been shown to be implicated in tumor formation, progression, metastasis, and drug resistance of various human cancers (16). For example, circRNA WHSC1 targets the miR-646/NPM1 pathway to promote the development of endometrial cancer (17); circRNA hsa_circRNA_104348 promotes hepatocellular carcinoma progression through modulating the miR-187-3p/RTKN2 axis and activating the Wnt/β-catenin pathway (18); circRNA_100367 regulated the radiation sensitivity of esophageal squamous cell carcinomas through the miR-217/Wnt3 pathway (19). In addition, because of the special closed loop structure of circRNAs, they hold a more stable construction as compared with most other linear RNAs; thus, they can be used as biomarkers to effectively diagnose and treat cancers (20). In lung cancer, a group of circRNAs have been found to be significantly dysregulated and involved in carcinogenesis and progression (19, 21). For instance, circRNA_103615 contributes to tumor progression and cisplatin resistance in NSCLC (22); circ-CPA4 regulates cell growth, stemness, drug resistance, and immune evasion in NSCLC (23); circP4HB promotes NSCLC aggressiveness and metastasis (24). Although the molecular mechanisms of these dysregulated circRNAs have been extensively investigated, the circRNA-activated oncogenic pathways that participate in NSCLC progression are still poorly understood.

Matrix metalloproteinase-9 (MMP-9), a significant matrix proteinase, is tightly regulated and expressed at low levels in normal physiological conditions, while it could be secreted by various cancer cells and plays vital roles in the progression of human malignant tumors, such as oral cancer, gastric cancer, lung cancer, liver cancer, breast cancer, and cervical cancer (25). Accumulating evidence has demonstrated that tumor-derived MMP-9 may destroy these tissue barriers and enhance the invasion and metastasis of tumor cells via cleaving extracellular matrix (ECM) proteins, which regulates ECM remodeling, and then affects invasion, metastasis, and angiogenesis of tumors (26). Thus, MMP-9 is considered as an oncogenic driver that affects the advancement of various carcinomas and is also a potential diagnostic or prognostic biomarker in several types of cancers (27, 28). In NSCLC, MMP-9 was discovered to be upregulated, and its overexpression could directly promote NSCLC metastasis (29). Nevertheless, whether MMP-9 might be regulated by circRNA in NSCLC has not been explored before. circDENND4C was an oncogene identified in breast cancer with a high level and was found to promote cell proliferation (30, 31). Moreover, there is a study that found that circDENND4C was also highly expressed in lung cancer (32), but it was not known whether circDENND4C boosted MMP-9 expression to modulate malignant behaviors of NSCLC cells. Based on the above studies, we speculated whether there was a potential relationship between circDENND4C and MMP-9. Then, we use the principle of circRNA acting on mRNA to search for a miRNA linking (14). circDENND4C had been predicted to be the regulator of miR-200b by bioinformatics, and increasingly, MMP-9 had been reported to be a target gene of miR-200b. Hence, further research is still needed to clarify the whole regulatory networks of circDENND4C and its downstream miR-200b and MMP-9 during the pathogenesis of lung cancer.

In this research, we measured circDENND4C, miR-200b, and MMP-9 abundance in NSCLC cell lines, and analyzed the function of circDENND4C, miR-200b, and MMP-9 contributing to lung cancer progression *in vitro*. Moreover, the interaction networks of circDENND4C, miR-200b, and MMP-9 were also demonstrated. To conclude, our work broadened our knowledge of NSCLC pathogenesis and provided potential therapeutic agents for this disease.

## 2 Materials and methods

### 2.1 Cell culture

The human normal bronchial epithelioid cells (HBE) and five NSCLC cell lines (PC9, A549, Calu-3, H1299, and SK-MES-1) were purchased from the Shanghai Institute of Cell Biology, Chinese Academy of Sciences (Shanghai, China). Among these different NSCLC cell lines, PC9, A549, Calu-3, and H1299 are adenocarcinomas, while SK-MES-1 is a squamous carcinoma. All cell lines were maintained in Dulbecco’s Modified Eagle Medium (DMEM; Hyclone, USA) containing 10% fetal bovine serum (FBS; Corning, USA), 100 units/mL penicillin, and 100 μg/mL streptomycin (Gibco, USA) under the standard conditions with 5% CO_2_ atmosphere with 37°C.

The detailed process of subsequent experiments is shown in **Supplementary Figure S1**. The expression of circDENND4C, miR-200b, and MMP-9 via quantitative real-time polymerase chain reaction (qRT-PCR) or Western blot in HBE and five NSCLC cell lines was firstly examined. Cells with significant changes in these three molecules in the NSCLC cell lines were selected for subsequent functional or mechanistic validation. The target relationships among circDENND4C, miR-200b, and MMP-9 were assessed by RNA fluorescence *in situ* hybridization (RNA-FISH), immunofluorescence (IF), dual-luciferase reporter system, qRT-PCR, and Western blot. Then, a cell count kit-8 (CCK-8) experiment, flow cytometry, and migration/invasion assays were performed to explore the role of circDENND4C, miR-200b, and MMP-9 in cell proliferation, cell cycle, apoptosis, cell migration, or invasion by transfecting with their overexpression or knockout plasmids. Finally, the influence of these three molecules on proteins related to cell adhesion and tight junction was further tested by Western blot and IF.

### 2.2 qRT-PCR

Cells were lysed in Trizol reagent (Invitrogen, USA) for the isolation of total RNA according to the manufacturer’s instructions. However, for extraction of circRNA, the total RNA was further enriched and pre-treated with RNase R enzyme (3 U/μg; Geneseed, China) for 20 min at 37°C, and subsequently purified by the RNeasy MinElute Cleaning Kit (Qiagen, USA). The quality and concentration of the purified total RNAs were detected using a NanoPhotometer N50 (Thermo Fisher, USA). Then, the complementary DNA (cDNA) was generated by reverse transcription using the First-Strand cDNA Synthesis Kit (Yeasen, Shanghai, China). Eventually, qRT-PCR was conducted via utilizing the mixture with SYBR (Thermo Fisher, USA) and specific primers. PCR conditions on the Gentier 96 system were as follows (TIANLONG, China): 95°C, 10 min, 1 cycle; 95°C, 15 s, 60°C, 60 s, 40 cycles; 95°C, 15 s, 60°C, l min, 95°C, 15 s (melting curve analysis). The special primers used in the current experiments were designed and purchased from Sangon Biotech (Shanghai, China), which are listed in **Table 1**. The glyceraldehyde-3-phosphate dehydrogenase (GAPDH) and U6 were regarded as the endogenous references and the 2^−ΔΔCt^ method was used to calculate the relative gene expression level.

**Table 1:** The primers used in this research with qRT-PCR.

Genes	Sequences
circDENND4C	Forward primer: 5′-GGGGCAGCAGTATTGTGAAA-3′ Reverse primer: 5′-AAGACTGTGTGCTCCCCATT-3′
miR-200b	Forward primer: 5′-GCGGCTAATACTGCCTGGTAA-3′ Reverse primer: 5′-GTGCAGGGTCCGAGGT-3′
U6	Forward primer: 5′-AAAGCAAATCATCGGACGACC-3′ Reverse primer: 5′-GTACAACACATTGTTTCCTCGGA-3′
MMP-9	Forward primer: 5′-TTGACAGCGACAAGAAGTGG-3′ Reverse primer: 5′-GAAGTTCACGTCGTCCTTAT-3′
GAPDH	Forward primer: 5′-GGCTGAGAACGGGAAGCTTGTCAT-3′ Reverse primer: 5′-CAGCCTTCTCCATGGTGGTGAAGA-3′

### 2.3 Western blot

The protein was prepared using a total protein extraction kit (Solarbio, China), and protein concentration was determined by a BCA protein assay kit (Beyotime, China), followed by denaturation at 98°C for 10 min. Equal amounts of proteins were subsequently subjected to sodium dodecyl sulfate-polyacrylamide gel electrophoresis (SDS-PAGE) and the proteins was transferred using polyvinylidene fluoride (PVDF) membranes (Millipore, USA). After the blockage of nonspecific binding sites using the Western blocking buffer (Beyotime, China), the membranes were incubated with primary antibodies and the corresponding secondary antibodies, and the dilutions of all antibodies were performed as recommended in their instructions (listed in **Table 2**). Finally, the protein bands were visualized via an enhanced chemiluminescence reagent (Beyotime, China). The gray values were analyzed by the QuantityOne software to evaluate relative protein levels and normalized to GAPDH.

**Table 2:** Detailed information about the reagent brand, Cat number, and diluted concentration of antibodies used in this study.

Name of antibody	Reagent brand	Cat number	Diluted concentration
MMP-9	Affinity, USA	AF5228	1:1,000 (Western blot); 1:200 (IF)
Occludin	Affinity, USA	DF7504	1:2,000 (Western blot); 1:200 (IF)
Claudin 5	Affinity, USA	AF5216	1:1,000 (Western blot); 1:200 (IF)
E-Cadherin	Affinity, USA	BF0219	1:1,500 (Western blot); 1:200 (IF)
Zonula occludens-1 (ZO-1)	Affinity, USA	AF5145	1:1,000 (Western blot); 1:200 (IF)
GAPDH	Abbinke, China	ABL1020	1:5,000 (Western blot)
Goat anti-rabbit IgG, HRP horseradish peroxidase labeled	Abbinke, China	A21020	1:10,000 (Western blot)
Goat anti-mouse IgG, HRP horseradish peroxidase labeled	Abbinke, China	A21010	1:10,000 (Western blot)
Goat anti-rabbit IgG, red DyLight 649 fluorescent marker	Abbinke, China	A23420	1:300 (IF)

### 2.4 RNA-FISH examination

The RNA-FISH hybridization kit of RiboBio (Guangzhou, China) was used for the experiment according to the instructions. A549 cells attached to slides were immobilized with 4% paraformaldehyde for 24 h. PBS buffer of 0.1% Triton X-100 was used for permeation at 4°C for 15 min. Prehybridization solution was added at 37°C for 30 min. Fluorescence-labeled hybridization probe and buffer were dripped onto the cell slide overnight at 37°C. Then, 42°C citric acid buffer was used for washing, and DAPI was added for nucleation with 5 min. Images were acquired using a laser confocal microscope (Leica, Germany).

### 2.5 IF staining

A549 cells attached to slides were fixed with 4% paraformaldehyde for 30 min. After that, cells were subjected to permeabilization by 0.5% Triton X-100 at 37°C for 15 min and blocked with 3% BSA at 37°C for 15 min to reduce the nonspecific binding. Then, cells were incubated with primary antibodies at 4°C overnight. After washing three times with PBS, the cells were further incubated with fluorescent secondary antibody at room temperature for 1 h. Finally, PBS was adopted to rinse the cells three times and DAPI was taken to stain them at 37°C for 5 min. Images were obtained with a laser confocal microscope (Leica, Germany).

### 2.6 Dual-luciferase reporter assay

The potential binding sites of miR-200b and circDENND4C or MMP-9 were predicted by starBase (http://starbase.sysu.edu.cn/index.php) or TargetScan (http://www.targetscan.org/vert_72/). circDENN4C sequence and MMP-9 3′-UTR containing wild-type (WT) or mutant-type (Mut) binding site for miR-200b were constructed into pGL3 luciferase vector (Promega, USA) to obtain circDENN4C-WT, circDENN4C-Mut, MMP-9-3′UTR-WT, and MMP-9-3′UTR-Mut. For the dual-luciferase reporter assay, A549 cells were transfected with the constructed luciferase reporter vectors and miR-200b mimic or miR-NC along with pRL-TK vector (Promega, USA). At 24 h post-transfection, the luciferase activities of firefly and renilla were measured with a dual-luciferase assay system (Promega, USA) following the instruction of the manufacturer.

### 2.7 Plasmid transfection

The overexpression and knockdown vectors of circDENN4C, miR-200b, and MMP-9 and the corresponding negative control were designed and constructed by Sangon Biotech (Shanghai, China). Lipofectamine 2000 (Invitrogen, USA) was employed to transfect the abovementioned plasmids into A549 cells. Forty-eight hours after the transfection, we harvested cells for detection of transfection efficiency by qRT-PCR. Meanwhile, the downstream molecules of circDENN4C were further examined to verify the interaction among circDENN4C, miR-200b, and MMP-9. Furthermore, a subsequent study was conducted to investigate the biological function of circDENN4C, miR-200b, and MMP-9 under the transfection of these overexpression and knockdown vectors.

### 2.8 Cell viability examination

The cells were collected and cell viability was measured by using the commercial CCK-8 kit (Beyotime, China) based on the protocol provided by the manufacturer. In brief, we plated A549 cells in 96-well plates at 2 × 10^4^ cells/well and let them grow in 10% FBS medium for 24 h. Following the transfection, each well was added with 10 μL of CCK-8 solution and cells underwent 2 h of incubation at 37°C in an incubator with 5% CO_2_. A spectrophotometer (Bio-Rad, USA) helped to read each well’s absorbance at 450 nm.

### 2.9 Cell proliferation capacity detection

5(6)-CFDA-SE is a cell tracer dye that can fluorescently label living cells for *in vitro* experiments on cell proliferation. According to the instructions of the CFDA SE cell proliferation and cell tracking kit (Abbinke, China), A549 cells were stained with CFDA SE staining solution away from light and then seeded into six-well plates. After transfection with the abovementioned vectors, cells were harvested at 48 h. The cell proliferation capacity was determined by a flow cytometer (Agilent, China) and results were analyzed using Novoexpress software.

### 2.10 Flow cytometry assay for cell cycle and apoptosis

Flow cytometry was used to assess cell cycle and apoptosis with the Cell Cycle and Apoptosis Analysis Kit (Beyotime, China). Briefly, approximately 2 × 10^5^ A549 cells were seeded into a 12-well plate and transfected with the abovementioned vectors. For the cell cycle, cells were collected and fixed with 70% alcohol overnight at −20°C. Afterwards, the staining buffer was added to resuspend the cells. Then, propidium iodide (PI) staining solution was added to the resuspended cells for 1 min at 37°C with no light. The DNA content was determined using flow cytometry (Agilent, China) and analyzed by Novoexpress software.

For apoptosis, cells were collected and resuspended in a binding buffer and stained in the dark with 5 μL of Annexin V–fluorescein isothiocyanate (FITC) and PI for 10 min. The cell death rate was eventually determined via a flow cytometer (Agilent, China), and results were analyzed using Novoexpress software.

### 2.11 Transwell assay

The 24-well plates with transwell inserts (Corning, USA) were used to test the abilities of migration and invasion. The inserts were precoated with Matrigel for the invasion assay, and the inserts that were not treated were used for the migration assay. After transfection, 3 × 10^5^ cells/mL cell suspensions of A549 were prepared in serum-free DMEM and 100-μL cell suspensions were plated into the top chambers, while DMEM containing 10% FBS was added to the lower chamber. After incubation for 24 h, a cotton swab was used to remove cells on the transwell member’s upper surface gently and the migrated or invasive cells on the transwell member’s lower surface were fixed with methanol and then stained with crystal violet for 20 min, followed by observation with three random fields under a 40× magnification microscope.

### 2.12 Statistical analysis

The experiments were performed at least three times. The statistical analysis was processed by GraphPad Prism 5, and the resulting data were presented as means ± standard error of the mean (SEM). An unpaired *t*-test was used for comparison between the two groups, whereas a comparison of the mean values between multiple groups was analyzed by one-way analysis of variance (ANOVA). Meanwhile, when the results of ANOVA were statistically significant, we further conducted *post-hoc* test comparisons, that is, via Dunnett’s multiple comparison test method, which is used to compare the differences between each group and the control group. Statistical significance was set at *p*-value < 0.05.

## 3 Results

### 3.1 The abnormal expression of circDENND4C, miR-200b, and MMP-9 in NSCLC cell lines

**Figure 1:**
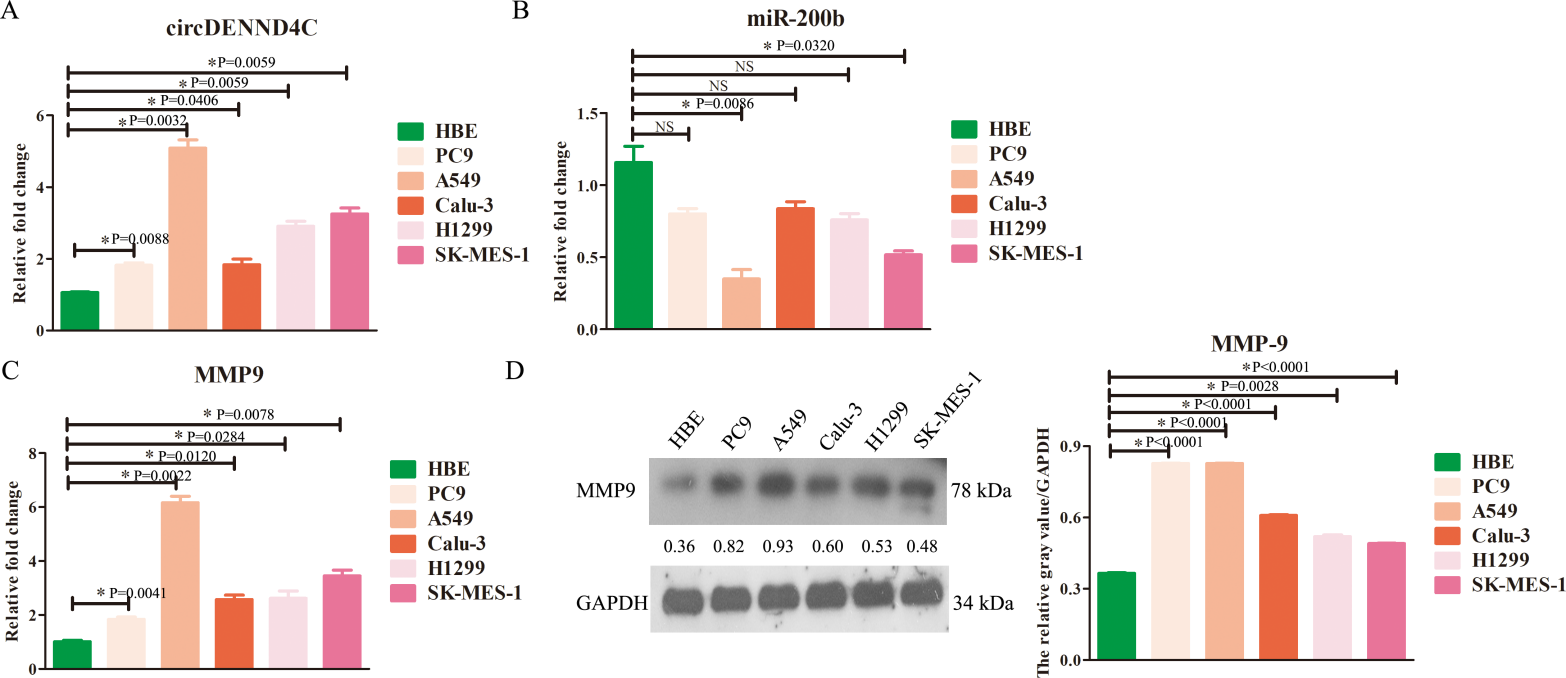
The changes of circDENND4C, miR-200b, and MMP-9 expression in NSCLC cell lines. **(A)** circDENND4C abundance was determined via qRT-PCR in HBE and five NSCLC cell lines. **(B)** miR-200b level was determined via qRT-PCR in HBE and five NSCLC cell lines. **(C)** The mRNA expression of MMP-9 was determined via qRT-PCR in HBE and five NSCLC cell lines. **(D)** The protein expression of MMP-9 was determined via qRT-PCR in HBE and five NSCLC cell lines. The gray values of the three biological repeats are also statistically represented by a bar chart. The data are presented as the mean ± SEM. NS, not significant. *p < 0.05 and the detailed *p*-values have been labeled in the graph.

qRT-PCR assay was initially conducted aiming at characterizing the expression of circDENND4C, miR-200b, and MMP-9 in NSCLC cell lines. The results showed that the expression of circDENND4C (**Figure 1A**) or MMP-9 (**Figure 1C**) was significantly upregulated in all NSCLC cell lines, while the expression of miR-200b (**Figure 1B**) was significantly downregulated in some NSCLC cell lines, especially A549 and SK-MES-1, as compared to the normal HBE cells. Further Western blot results also validated that the protein expression of MMP-9 exhibited a relatively higher level in NSCLC cell lines than that in HBE cells (**Figure 1D**), which was in accordance with the qRT-PCR results. Thus, these data suggested that circDENND4C, miR-200b, and MMP-9 presented abnormal expression in NSCLC cell lines and might be potential prognostic biomarkers in NSCLC.

Moreover, it was obviously seen that the expression of circDENND4C, miR-200b, and MMP-9 in A549 cells was most significant in A549 cells compared to the other NSCLC cell lines; thus, A549 cells were chosen for the following research.

### 3.2 circDENND4C enhances MMP-9 expression via absorbing miR-200b

**Figure 2:**
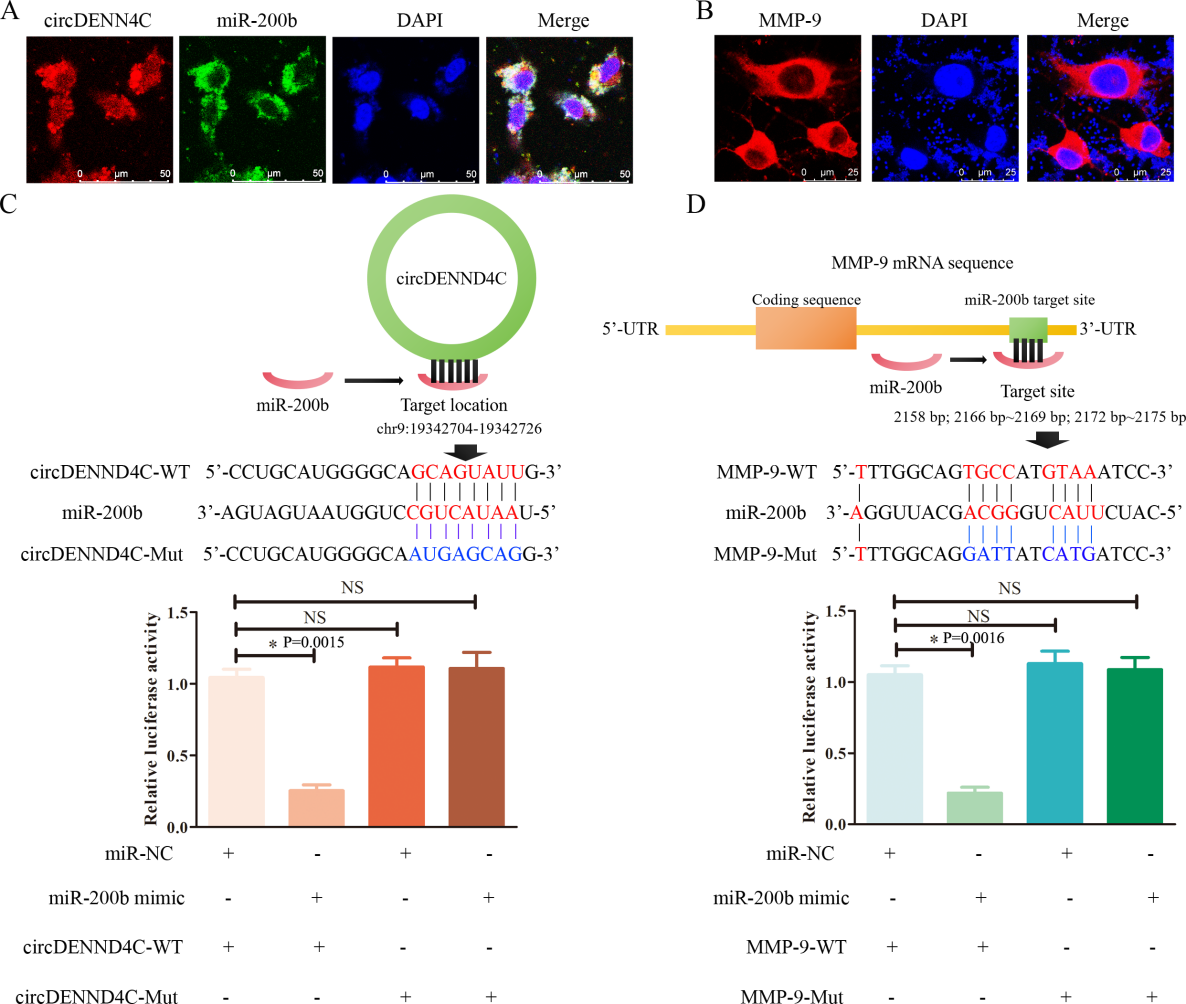
The target associations among circDENND4C, miR-200b, and MMP-9. **(A)** Subcellular co-localization of circDENND4C and miR-200b detected by RNA-FISH. **(B)** IF for subcellular localization of MMP-9. **(C)** The model map showed the binding sites between circDENND4C and miR-200b. The predicted WT and Mut circDENND4C binding site in miR-200b with marking in red and blue, respectively. Relative luciferase activities were examined in A549 cells co-transfected with circDENND4C-WT or circDENND4C-Mut and miR-200b mimics or miR-NC by dual luciferase reporter gene assay. **(D)** The model map showed the binding sites between miR-200b and MMP-9. The predicted WT and Mut MMP-9 binding sites in miR-200b with marking in red and blue, respectively. Relative luciferase activities were examined in A549 cells co-transfected with MMP-9-WT or MMP-9-Mut and miR-200b mimics or miR-NC by dual luciferase reporter gene assay. The data are presented as the mean ± SEM. NS: not significant. *p < 0.05 and the detailed *p*-values have been labeled in the graph.

It is well known that circRNA can act as a ceRNA to absorb miRNA and indirectly stimulate the expression of target genes of miRNAs (33, 34). Using online tools, we found that miR-200b may have a high probability of binding to circDENND4C (**Figure 2C**), and MMP-9 also had complementary binding sequences with miR-200b (**Figure 2D**). Furthermore, it was shown that circDENND4C was mainly distributed in the cytoplasm and co-localized with miR-200b (**Figure 2A**), suggesting that circDENND4C may exhibit its function via acting on miR-200b. Meanwhile, MMP-9 was also discovered to be localized in the cytoplasm (**Figure 2B**). Subsequently, a luciferase reporter gene assay was carried out to further verify the targeted relationship between circDENND4C and miR-200b, as well as between miR-200b and MMP-9. It was discovered that the luciferase activity was evidently reduced by transfection of miR-200b mimics in the circDENND4C-WT group, while it was not changed in the circDENND4C-Mut group (**Figure 2C**), supporting the direct interaction between circDENND4C and miR-200b. Additionally, it was also observed that the luciferase activity was notably inhibited by the miR-200b mimics in the MMP-9-WT group, but it was not changed by the miR-200b mimics in the MMP-9-Mut group (**Figure 2D**), indicating that MMP-9 served as a direct target of miR-200b.

**Figure 3:**
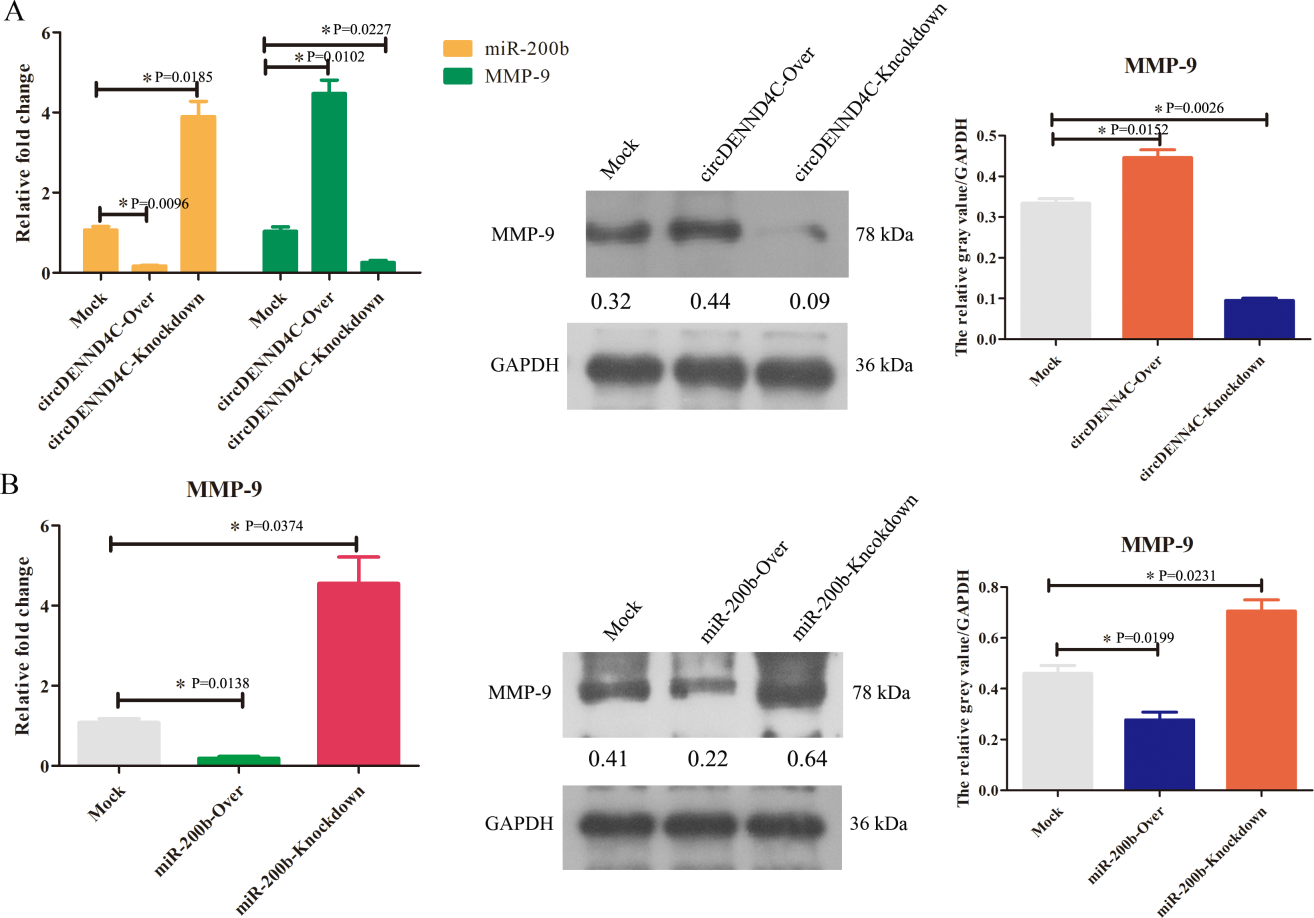
The regulatory relationship among circDENND4C, miR-200b, and MMP-9. **(A)** MMP-9 mRNA and protein expressions in circDENND4C-knockdown and overexpressing transfected A549 cells detected by qRT-PCR and Western blot, respectively. **(B)** MMP-9 mRNA and protein expressions in miR-200b-knockdown and overexpressing transfected A549 cells detected by qRT-PCR and Western blot, respectively. The data are presented as the mean ± SEM. *p < 0.05 and the detailed *p*-values have been labeled in the graph.

Moreover, the overexpression or knockout vectors of these three molecules have been obtained and the transfection efficiency of these vectors has been proven to be effective (**Supplementary Figure S2**). Subsequently, qRT-PCR results further revealed that circDENND4C overexpression suppressed miR-200b expression (**Figure 3A**), and miR-200b overexpression suppressed MMP-9 expression (**Figure 3B**); meanwhile, circDENND4C knockdown promoted miR-200b expression (**Figure 3A**), and miR-200b knockdown promoted MMP-9 expression (**Figure 3B**). Thus, there was indeed a negative correlation between circDENND4C and miR-200b, as well as between miR-200b and MMP-9. Moreover, because circRNAs act as miRNA sponges to block or reduce miRNA expression and then promote the expression of target mRNA, we then sought to determine whether circDENND4C regulated MMP-9 expression. It was found that overexpression of circDENND4C could enhance the mRNA and protein expressions of MMP-9, but knockdown of circDENND4C inhibited the mRNA and protein expressions of MMP-9 (**Figure 3A**), which implied that circDENND4C promoted MMP-9 expression probably by sponging miR-200b.

### 3.3 The circDENND4C/miR-200b/MMP-9 axis regulates lung cancer cell growth, cell cycle, apoptosis, migration, and invasion

**Figure 4:**
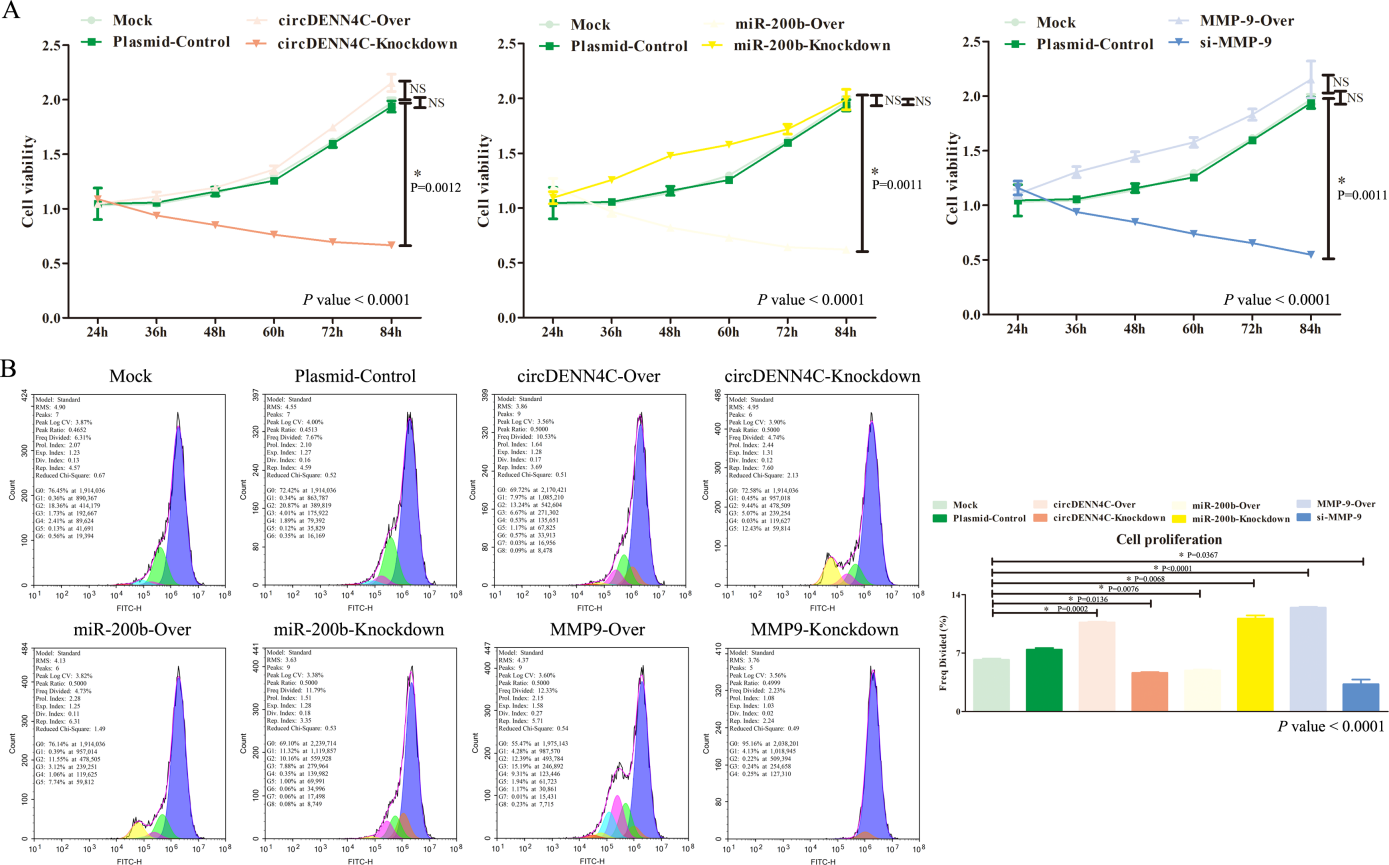
Effects of the circDENND4C/miR-200b/MMP-9 regulatory axis on cell viability and cell proliferation of A549 cells. **(A)** Cell viability was measured by the CCK-8 assay. **(B)** Cell proliferation was measured by the CFSE assay. The data are presented as the mean ± SEM. The *p*-values at the bottom right of the line chart and bar chart are obtained by ANOVA among multiple groups, while the NS and detailed *p*-values labeled in the line chart and bar chart are the results of post-ANOVA comparison. NS, not significant.

Afterward, we started to explore the potential biological function of circDENND4C, miR-200b, and MMP-9. Considering that the changes of the circDENND4C/miR-200b/MMP-9 regulatory axis in A549 cells were the most significant, we chose A549 cells for relevant verification in the following functional studies. Firstly, we measured cell viability through the CCK-8 assay. The data displayed that although circDENND4C overexpression, miR-200b inhibition, and MMP-9 overexpression did not significantly improve cell viability in A549 cells, a slight upregulation can be seen from **Figure 4A**, while circDENND4C knockdown, miR-200b overexpression, and MMP-9 silence distinctly suppressed cell proliferation in A549 cells (**Figure 4A**). Given the metabolic activity of cells measured in the CCK-8 experiment, which is directly proportional to the number of viable cells in the culture, the increased metabolic activity does not always directly correlate with increased cell number, as some treatments might affect cellular metabolism without changing cell numbers. Thus, we further employed CFDA-SE reagents to detect cell proliferation. The data showed that circDENND4C overexpression, miR-200b inhibition, and MMP-9 overexpression significantly promoted cell proliferation based on the higher-frequency division, whereas circDENND4C knockdown, miR-200b overexpression, and MMP-9 silence significantly suppressed cell proliferation based on the lower-frequency division (**Figure 4B**).

**Figure 5:**
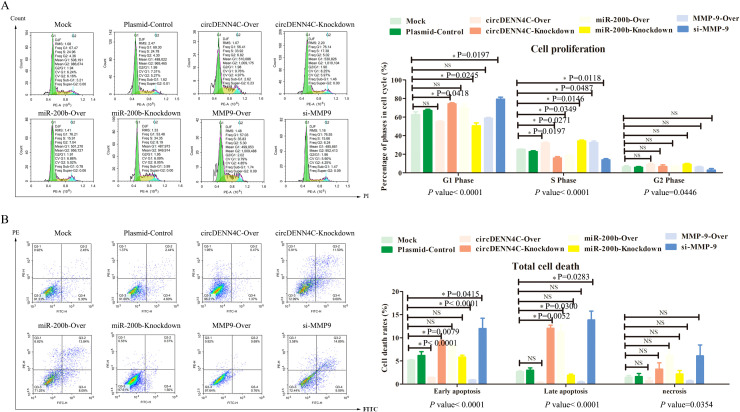
Influences of the circDENND4C/miR-200b/MMP-9 regulatory axis on cell cycle and cell death of A549 cells. **(A)** Flow cytometry was used to analyze the cell cycle in A549 cells transfected with the overexpression and knockdown vectors of circDENN4C, miR-200b, and MMP-9. **(B)** Flow cytometry was used to analyze the apoptotic rate of A549 cells transfected with the overexpression and knockdown vectors of circDENN4C, miR-200b and MMP-9.The data are presented as the mean ± SEM. The *p*-values at the bottom of the bar chart are obtained by ANOVA among multiple groups, while the NS and detailed *p*-values labeled in the bar chart are the results of post-ANOVA comparison. NS, not significant.

Subsequently, the effects on cell cycle and apoptosis were examined by flow cytometry. Different phases of cell cycle have different meanings. The G1 phase of the cell cycle represents the early phase of DNA synthesis, the S phase of the cell cycle represents the DNA synthesis phase, and the G2 phase of the cell cycle represents the late phase of DNA synthesis; thereby, our focus was mainly on the S phase, which indirectly determined whether the cell can quickly move into the next cycle of proliferation. The flow cytometry analysis disclosed that circDENND4C overexpression, miR-200b inhibition, and MMP-9 overexpression led to more cells arrested in the S phase, but transfecting with the vectors of circDENND4C knockdown, miR-200b overexpression, and MMP-9 silence resulted in a relatively lower cell percentage in the S phase, and there was almost no significant difference in the G1 phase and G2 phase of each group (**Figure 5A**). Apoptosis is a programmed mode of death, but a key feature of cancer cells is that they do not perform death, but grow rapidly; therefore, apoptosis examination is usually performed to observe the characteristics of cancer cells. As illustrated in **Figure 5B**, Q3-1 represents necrotic cells, Q3-2 represents late apoptotic cells, and Q3-4 represents early apoptotic cells. The flow cytometry data disclosed that there were no significant differences in necrosis among all groups. Meanwhile, it was clearly seen that the overexpression of circDENND4C and MMP-9 triggered a marked decrease in early apoptosis of A549 cells, but the silence of circDENND4C and MMP-9 triggered a marked increase in the early apoptosis of A549 cells (**Figure 5B**). Additionally, circDENND4C knockdown, miR-200b overexpression, and MMP-9 silence induced a remarkable increase in the late apoptosis of A549 cells, but circDENND4C overexpression, miR-200b knockdown, and MMP-9 overexpression induced a relative decrease in the late apoptosis of A549 cells (**Figure 5B**).

**Figure 6:**
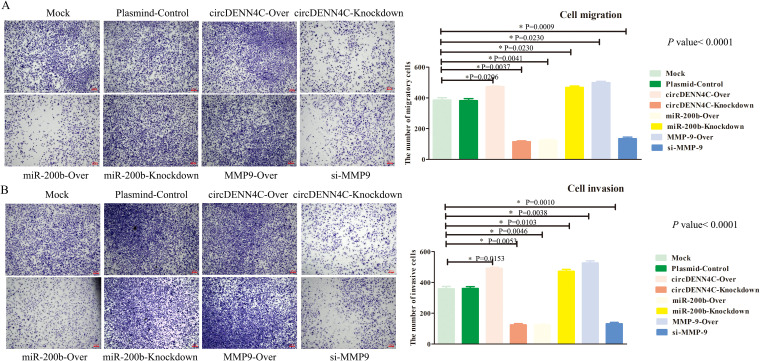
The circDENND4C/miR-200b/MMP-9 axis regulated NSCLC cell migration and invasion. **(A)** The migration of A549 cells transfected with the indicated vectors was assessed by the transwell migration assay. **(B)** The invasion of A549 cells transfected with the indicated vectors was assessed by transwell invasion assay. The data are presented as the mean ± SEM. The *p*-values on the right side of the bar chart are obtained by ANOVA among multiple groups, while the detailed *p*-values labeled in the bar chart are the results of post-ANOVA comparison. NS, not significant.

Finally, we continued to investigate the role of circDENND4C, miR-200b, and MMP-9 in metastasis via the transwell assay. The results showed that circDENND4C overexpression, miR-200b inhibition, and MMP-9 overexpression gave rise to increased A549 cell migration and invasion. In contrast, the number of migrating and invading cells was much less in circDENND4C knockdown, miR-200b overexpression, and si-MMP-9 groups (**Figure 6**). Collectively, the above findings pointed out that the circDENND4C/miR-200b/MMP-9 axis might significantly affect the biological behavior of lung cancer cells, including cell proliferation, cell cycle, apoptosis, migration, and invasion.

### 3.4 The circDENND4C/miR-200b/MMP-9 axis affects cell metastasis by altering cell adhesion and tight junction

**Figure 7:**
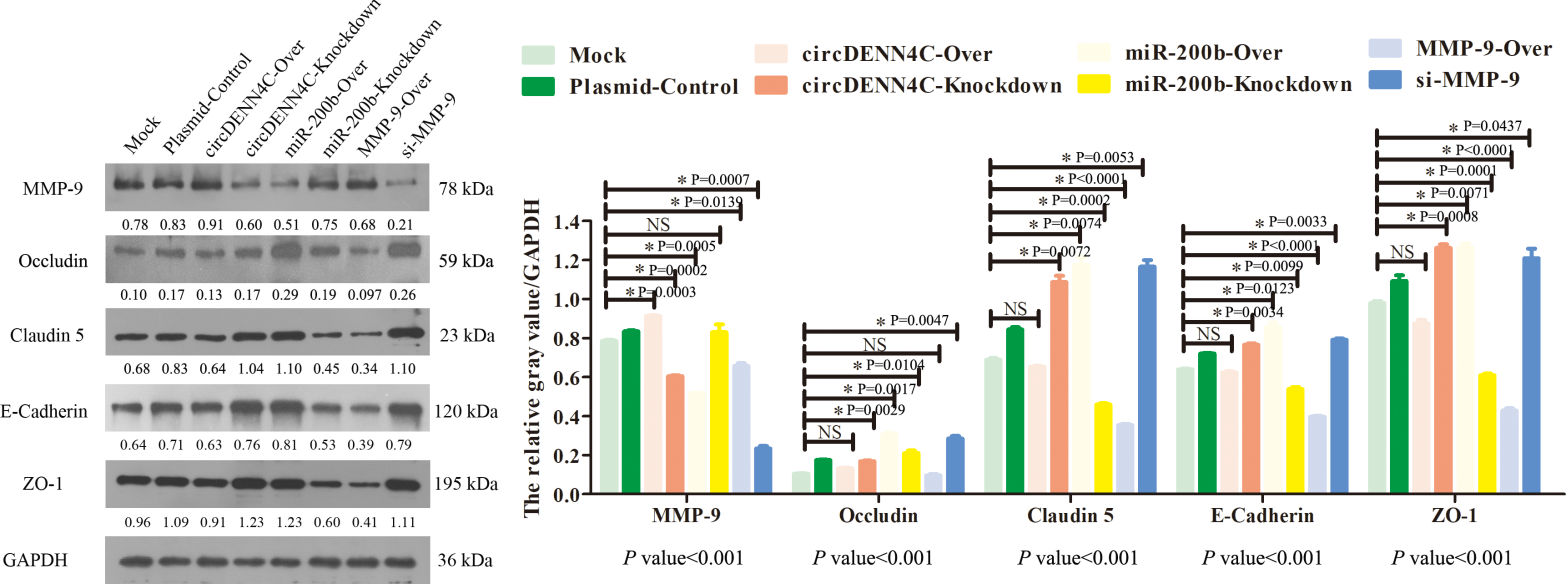
The levels of MMP-9, Occludin, ZO-1, Claudin 5, and E-cadherin proteins were tested by Western blot assay in the A549 cells transfected with the indicated vectors. The protein relative expression value was listed in the figure, which is equal to the protein gray value divided by the gray value of the internal reference protein GAPDH. The gray values of the three biological repeats are also statistically represented by a bar chart. The *p*-values at the bottom of the bar chart are obtained by ANOVA among multiple groups, while the NS and detailed *p*-values labeled in the bar chart are the results of post-ANOVA comparison. NS, not significant.

**Figure 8:**
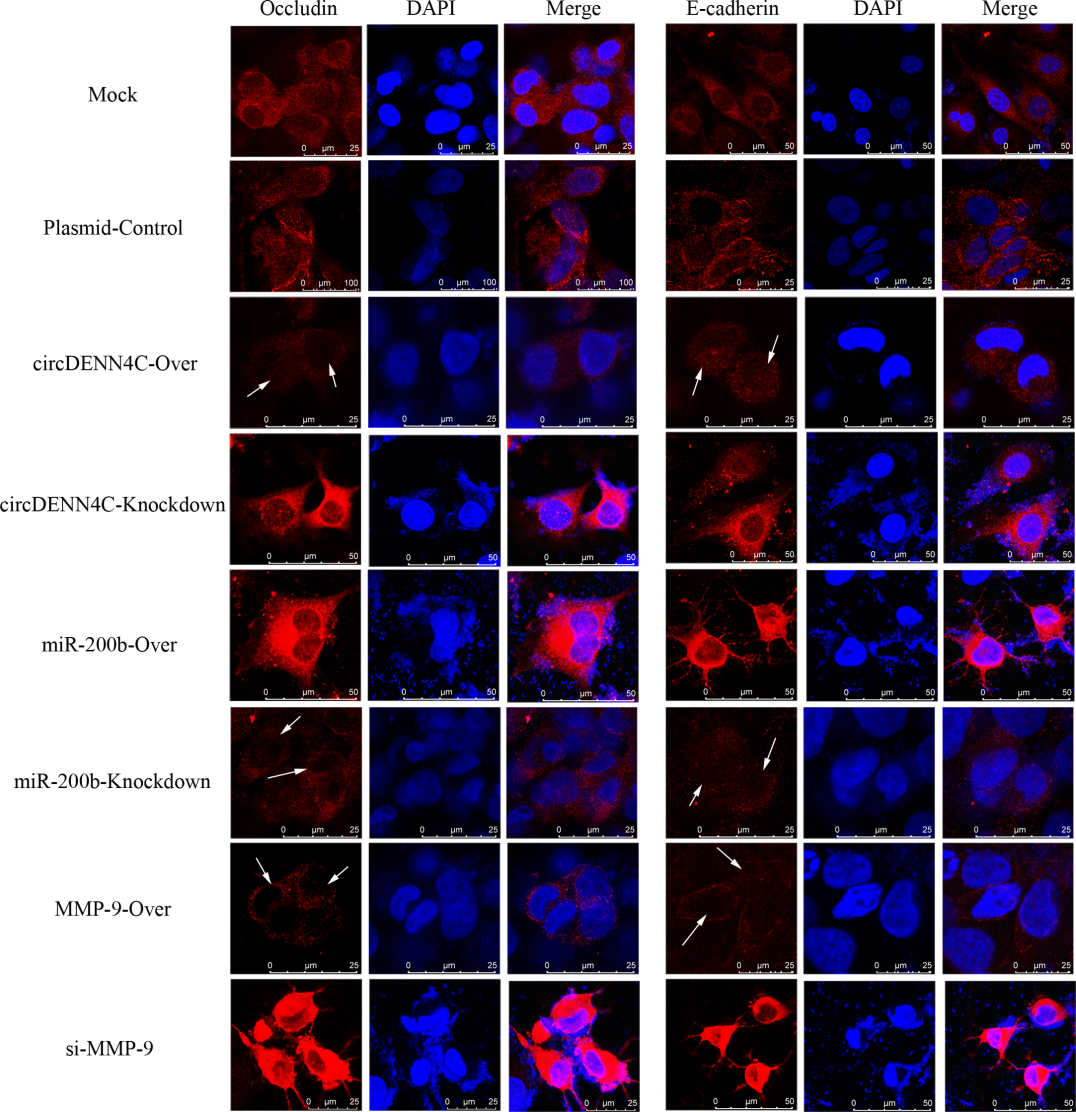
IF analysis was performed to observe the location and expression of Occludin and E-cadherin in A549 cells after transfection with the overexpression and knockdown vectors of circDENN4C, miR-200b, and MMP-9.

Previous studies have confirmed that the MMP-9 protein promotes tumor migration by degrading EMC or the linker complex between cells (25, 26). To determine the potential mechanism of the circDENND4C/miR-200b/MMP-9 regulatory axis in cell metastasis, some adhesion and tight junction proteins were further tested with Western blot experiments. It was obviously revealed that MMP-9 was elevated in circDENND4C overexpression, miR-200b knockdown, and MMP-9 overexpression groups, and the expression of Occludin, E-cadherin, Claudin 5, and ZO-1 was reduced in these groups (**Figure 7**). Additionally, the IF results also clearly exhibited the disruptions of cell junctions about Occludin and E-cadherin in these groups (**Figure 8**). Conversely, circDENND4C knockdown, miR-200b overexpression, and MMP-9 silence treatments caused increasing expression of Occludin, E-cadherin, Claudin 5, and ZO-1 (**Figure 7**). Moreover, the changes in Occludin and E-cadherin were further examined by IF (**Figure 8**), pointing that circDENND4C might change the metastatic ability of lung cancer cells via targeting the miR-200b/MMP-9 axis, further acting on the proteins of cell adhesion and tight junction.

## 4 Discussion

Lung cancer is the most common type of cancer, and approximately 85% of patients with lung cancer are known to have NSCLC, which is mainly attributed to uncontrolled tobacco consumption and increasing environmental pollution in China (35). Early diagnosis and treatment of patients with lung cancer can significantly prolong the survival of patients and improve their quality of life (1, 2). Thus, it is urgent to further explore the mechanisms of lung tumorigenesis and find sensitive, specific, and effective indicators for the diagnosis and treatment of lung cancer. In recent years, circRNAs, regarded as critical regulators in cancer progression, have attracted considerable attention and have been gradually discovered to be superior diagnostic markers and therapeutic targets for cancer to linear transcripts, because circRNA lacks a 5′-end cap structure and a 3′-poly A tail structure, which is resistant to hydrolysis by RNA exonuclease and is highly stable (16, 20). Increasing evidence has demonstrated that a dysregulated level of circRNA may function as antioncogenes or tumor promoters in lung cancer through competitive binding to miRNAs and inducing the functional dysregulation of miRNAs’ target genes (19, 36). For example, circRNA-6834 suppresses NSCLC progression by destabilizing ANHAK and regulating the miR-873-5p/TXNIP axis (37); CircHERC1 promotes NSCLC cell progression by sequestering FOXO1 in the cytoplasm and regulating the miR-142-3p-HMGB1 axis (38). This research aimed to explore the effects and molecular mechanisms of circDENND4C and its downstream factors in the occurrence and development of lung cancer, so as to provide a new marker for the early diagnosis and prevention of high-risk groups of lung cancer, while also offering a valuable potential therapeutic target for the treatment of lung cancer.

Previous studies have uncovered that circDENND4C has functions in a variety of tumors, such as colorectal cancer (39), breast cancer (31), epithelial ovarian cancer (40), and even lung cancer (32). Moreover, MMP-9, an important oncogene, belongs to the zinc-dependent endopeptidase family, can degrade ECM, and plays a vital role in tumorigenesis and metastasis, such as breast cancer (41), acute leukemia (42), pancreatic cancer (43), and even lung cancer (44). However, it is well-known that circRNAs often serve as sponges of miRNAs and are further involved in the regulation of miRNA target genes (14, 34). Thus, based on the above theories, we identified that miR-200b may be the bridge between circDENND4C and MMP-9 through bioinformatics analysis. Here, we mainly explored the effect of circDENND4C, miR-200b, and MMP-9 in the progression of lung cancer, and from the overall results of this study, it was found that (1) circDENND4C, miR-200b, and MMP-9 have significant changes in NSCLC cell lines (2); the sequential targeted regulatory relationship among circDENND4C, miR-200b, and MMP-9 was firstly confirmed; and (3) circDENND4C, miR-200b, and MMP-9 were involved in the changes of biological behavior of lung cancer cells, including cell proliferation, cell cycle, apoptosis, migration, and invasion. Thus, we can draw a key conclusion that circDENND4C accelerated lung cancer progression, and this effect was related to the miR-200b/MMP-9 axis. Then, we will discuss the results of this study one by one.

The role of circDENND4C, miR-200b, and MMP-9 in cancer progression has been extensively studied in previous studies. For instance, circDENND4C upregulates TCF4 expression to modulate hepatocellular carcinoma cell proliferation and apoptosis via activating the Wnt/β-catenin signal pathway (45); miR-200b suppresses gastric cancer cell migration and invasion by inhibiting NRG1 through ERBB2/ERBB3 signaling (46); the co-expression of MMP-9 and Tenascin-C is significantly associated with the progression and prognosis of pancreatic cancer (47). Moreover, the effects of circDENND4C, miR-200b, and MMP-9 in lung cancer have also been investigated separately. For instance, circDENND4C was found to promote proliferation and metastasis of lung cancer (32), miR-200b was also discovered to inhibit tumor growth and chemoresistance in lung cancer (39), and MMP-9 was further disclosed to potentiate lung cancer cell migration and invasion (44). However, a comprehensive analysis of the three molecules is not yet evident in cancer research, including lung cancer. In this study, consistent with previous studies, it was found that circDENND4C and MMP-9 were significantly upregulated in all NSCLC cell lines, but miR-200b was markedly downregulated in some NSCLC cell lines, in comparison with the normal HBE cells, which suggested that the abnormal expression of circDENND4C, miR-200b, and MMP-9 might be closely correlated with the progression of lung cancer, and may be a potential biomarker and therapeutic target for NSCLC.

However, the regulatory relationship among circDENND4C, miR-200b, and MMP-9 is still completely undefined. We used RNA-FISH and IF experiments to confirm that circDENND4C and miR-200b could co-locate in the cytoplasm, and MMP-9 was also expressed in the cytoplasm. Subsequently, the dual-luciferase report assay directly pointed out the targeting relationship between circDENND4C and miR-200b, as well as between miR-200b and MMP-9. Finally, we further confirmed the negative regulatory effect of circDENND4C on miR-200b, as well as that of miR-200b on MMP-9. In addition, it was found that circDENND4C promoted MMP-9 expression via sponging miR-200b, indicating the possible regulatory effect of circDENND4C on MMP-9 through miR-200b. However, accumulating evidence has uncovered the fact that circRNA could act as a sponge for miRNA to relieve the suppression of its target gene (48). Therefore, these findings pointed out that the regulatory effect of circDENND4C on MMP-9 was likely to be exerted indirectly through the effect of circDENND4C on miR-200b. Additionally, other studies have also confirmed the targeting relationship between circDENND4C and miR-200b (30), as well as the regulatory relationship between miR-200b and MMP-9 (49), which exactly matched what we found. Furthermore, it has been reported that miR-200 plays a key role in the invasion of lung cancer progression by targeting MMP-9 (50).

Subsequently, based on the results of the most significant changes in circDENND4C, miR-200b, and MMP-9 in NSCLC cell lines, we selected A549 cells for the follow-up studies on the biological function of circDENND4C, miR-200b, and MMP-9. Our data revealed that circDENND4C knockdown, miR-200b overexpression, and MMP-9 silence obviously suppressed lung cancer cell proliferation, arrested cell-cycle progression, and reduced cell migration and invasion *in vitro*, while promoting cell death, but circDENND4C overexpression, miR-200b inhibition, and MMP-9 overexpression significantly reversed the above results via the gain- and loss-of-function experiments. Our results were consistent with previous studies on the role of circDENND4C, miR-200b, and MMP-9 in cancer cells (26, 30, 32). Actually, characteristics typical of cells with a malignant phenotype include uncontrolled growth and proliferation, avoidance of apoptosis, insensitivity to anti-growth signals, continuous angiogenesis, self-sustained growth signals, tissue invasion, and metastasis (51). Moreover, metastasis is responsible for the greatest number of cancer-related deaths (52). Hence, in the present study, cell proliferation, cell cycle, apoptosis, migration, and invasion were also selected as indicators to observe the functional changes of cancer cells in the case of circDENND4C, miR-200b, and MMP-9 overexpression and knockdown. First of all, enhanced proliferation via replicative division in cancer cells means the rapid growth of cancer cells. Secondly, healthy cells typically undergo programmed cell death or apoptosis during the regular progression of the cell cycle, but cancer cells evade apoptosis to enter a new cell cycle for their growth. Lastly, metastasis is the leading cause of disease exacerbation or cancer treatment failure and death in cancer patients (51, 52). Thus, understanding the dynamic process of tumor cell growth and metastasis will help identify targets for molecular diagnosis or therapy. The results of these indicators in our study not only helped us to confirm that the circDENND4C/miR-200b/MMP-9 regulatory network existed in NSCLC, but also might indicate that knockdown of circDENND4C halted or possibly reversed NSCLC growth and metastasis by inhibiting MMP-9 with releasing miR-200b.

The maintenance of cell integrity is partially determined by the adhesion and tight junction between cells (53, 54). However, MMPs can exert the deteriorating effects on cell components due to the fact that MMPs function in the degradation and turnover of components of adhesion and tight junction between cells; therefore, it could increase cell permeability, disrupt cell structure, and destroy cell integrity (55, 56). In the MMPs family, the MMP-9 protein is found to exist in different types of cancer, and it is believed to facilitate tumor invasion, migration, and metastasis (25, 26). Thus, in this study, we continued to investigate whether the circDENND4C/miR-200b/MMP-9 regulatory axis was involved in mediating cell invasion and migration through altering cell adhesion and tight junction. The results presented that underexpression of circDENND4C and MMP-9, as well as overexpression of miR-200b, damaged tight and adhesion junctions’ components, and *vice versa*. In fact, accumulating evidence has already indicated that the cleavage of tight and adhesion junction proteins, such as Occludin and E-cadherin, in tumor cells supports cancer cell migration, invasion, and metastasis (57). Hence, our data implied that the circDENND4C/miR-200b/MMP-9 regulatory axis was shown to modulate the tight and adhesion junctions to control the metastasis of lung cancer cells.

**Figure 9:**
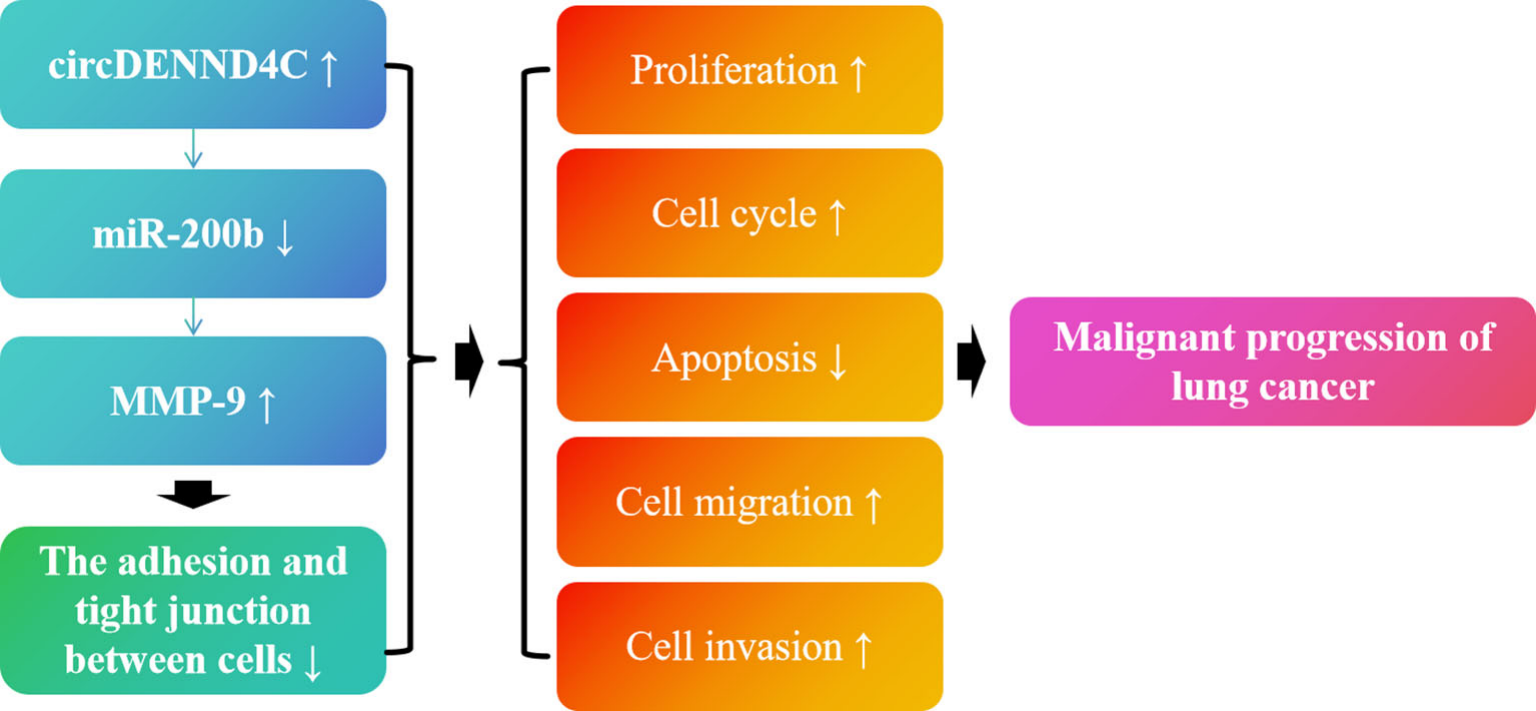
Schematic diagram of the role of the circDENND4C/miR-200b/MMP-9 regulatory axis in lung cancer progression.

To sum up, in the current study, we demonstrated that circDENND4C and MMP-9 are more highly expressed in NSCLC cell lines, while miR-200b is lowly expressed in NSCLC cell lines. Moreover, it was first confirmed that circDENND4C might sponge miR-200b to regulate MMP-9 expression. Additionally, our results further pointed out that circDENND4C accelerated lung cancer cell progression by causing the promotion of cell proliferation, cell cycle, migration, invasion, and inhibition of apoptosis, possibly via regulating miR-200b/MMP-9 axis-mediated damaged connections between cells (**Figure 9**). Nevertheless, it is undeniable that we only used A549 cells for validation *in vitro* experiments when conducting functional studies on the circDENND4C/miR-200b/MMP-9 regulatory axis and did not use other NSCLC cell lines or nude mouse animal models for validation. Thus, this study has limitations to a certain extent. However, from the results of our study, the role of the circDENND4C/miR-200b/MMP-9 regulatory axis in the progression of lung cancer cannot be ignored. Moreover, based on the cell-type-specific, tissue-specific, or developmental-stage-specific expression of circRNAs and their greater stability owing to their circular structure, circRNAs could be considered as biological markers of human diseases and improve the accuracy and specificity of diagnoses and therapies (58). Thus, our research may indicate a potential mechanism of action in lung cancer and provide a new theoretical basis for the prognosis and treatment of lung cancer. Although there have been some breakthroughs in circRNA research in recent years, there are still questions that need to be answered. In the future, we can not only further verify the downstream mechanism of circRNA at the *in vitro* level, but also pay attention to the upstream mechanism of circRNA.

## Data Availability

The original contributions presented in the study are included in the article/**Supplementary Material**. Further inquiries can be directed to the corresponding authors.

## Supplementary material

Supplementary Figure 1 The experimental operation flow chart of this study. The content in the blue box is the verification of the expression of circDENN4C, miR-200b and MMP-9 in NSCLC cell lines, the content in the green box is the verification of the plasmids of circDENN4C, miR-200b and MMP-9, and the content in the red box is the verification of the targeted regulatory interaction of circDENN4C, miR-200b and MMP-9, while the content in the black box is the verification of the biological function of circDENN4C, miR-200b and MMP-9. Black bold font is the methodology, while red bold font is the cell type selected for the targeted relationship validation and biological function validation experiments.

Supplementary Figure 2 Validation of plasmid transfection efficiency. qRT-PCR determined the levels of circDENND4C **(A)**, miR-200b **(B)** and MMP-9 **(C)** in A549 cells after the transfection. The data are presented as the mean ± SEM. **p* < 0.05 and the detailed *p*-values have been labeled in the graph.
