# Correction: circDENND4C serves as a sponge for miR-200b to drive non-small cell lung cancer advancement by regulating MMP-9 expression

**DOI:** 10.3389/fonc.2025.1641204

**Published:** 2025-09-02

**Authors:** Yaming Lv, Lan Wang, Yunhui Zhang, Dong Wei, Yajie Hu

**Affiliations:** ^1^ Department of Respiratory Medicine, The Affiliated Hospital of Kunming University of Science and Technology, Kunming, China; ^2^ Department of Respiratory Medicine, The First People’s Hospital of Yunnan Province, Kunming, China; ^3^ Department of Hepatopancreatobiliary Surgery, The Second Affiliated Hospital of Kunming Medical University, Kunming, China

**Keywords:** circDENND4C, miR-200b, MMP-9, non-small cell lung cancer (NSCLC), tumor progression

There was a mistake in **Figure 5B** and **Figure 6B** as published. In **Figure 5B**, due to the author’s unintentionally arranged error, the flow cytometry image of apoptosis results of the miR-200b-Knockdown group was incorrected as the image of the Mock group. In **Figure 6B**, due to the author adopted the same photoing techniques to obtain many high quality pictures for the migration and invasion experiments, the original picture sources of the Plasmid-Control group and the miR-200b-Over group were unintentionally misused when the author selected the photos and arranged them. The corrected [**Figure 5B** and **Figure 6B**] and its caption “**Figure 5B** Flow cytometry was used to analyze the apoptotic rate of A549 cells transfected with the overexpression and knockdown vectors of circDENN4C, miR-200b and MMP-9. The data are presented as the mean ± SEM.” and “**Figure 6B** The invasion of A549 cells transfected with the indicated vectors was assessed by transwell invasion assay. The data are presented as the mean ± SEM.” appear below.

**Figure 5:**
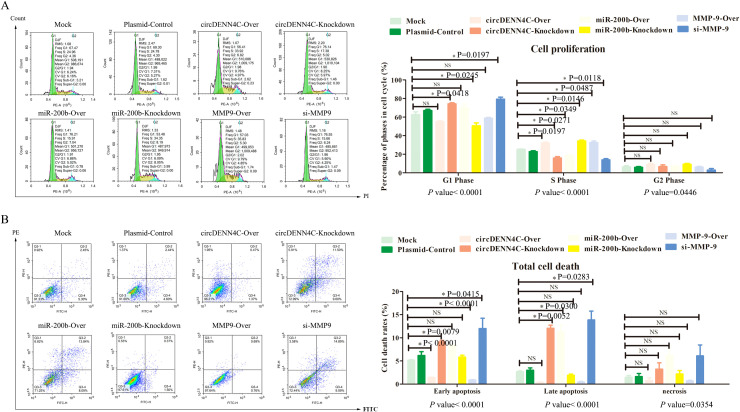
Influences of the circDENND4C/miR-200b/MMP-9 regulatory axis on cell cycle and cell death of A549 cells. **(A)** Flow cytometry was used to analyze the cell cycle in A549 cells transfected with the overexpression and knockdown vectors of circDENN4C, miR-200b, and MMP-9. **(B)** Flow cytometry was used to analyze the apoptotic rate of A549 cells transfected with the overexpression and knockdown vectors of circDENN4C, miR-200b and MMP-9.The data are presented as the mean ± SEM. The *p*-values at the bottom of the bar chart are obtained by ANOVA among multiple groups, while the NS and detailed *p*-values labeled in the bar chart are the results of post-ANOVA comparison. NS, not significant.

**Figure 6:**
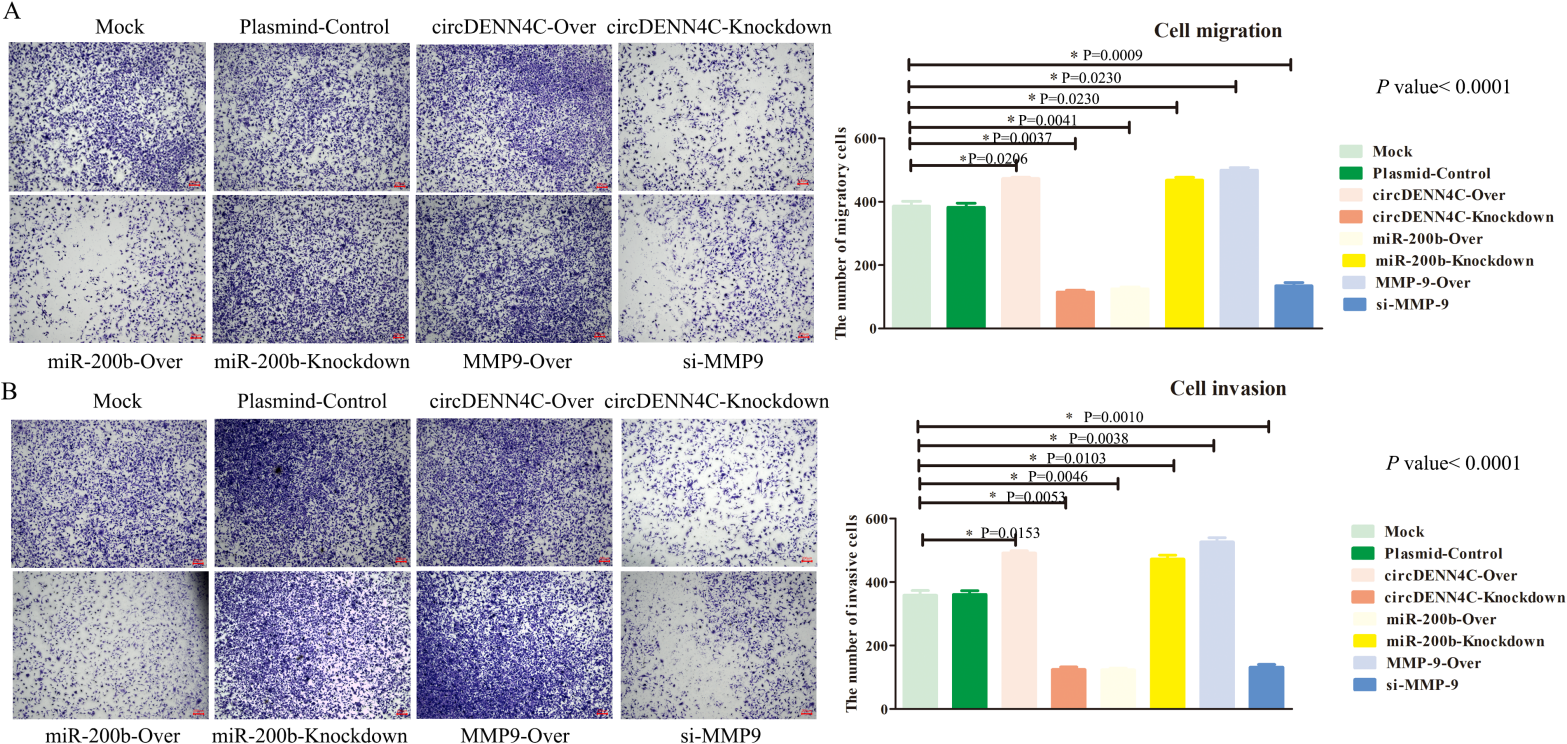
The circDENND4C/miR-200b/MMP-9 axis regulated NSCLC cell migration and invasion. **(A)** The migration of A549 cells transfected with the indicated vectors was assessed by the transwell migration assay. **(B)** The invasion of A549 cells transfected with the indicated vectors was assessed by transwell invasion assay. The data are presented as the mean ± SEM. The *p*-values on the right side of the bar chart are obtained by ANOVA among multiple groups, while the detailed *p*-values labeled in the bar chart are the results of post-ANOVA comparison. NS, not significant.

The original article has been updated.

