# Supplementary figures and images for: circDENND4C serves as a sponge for miR-200b to drive non-small cell lung cancer advancement by regulating MMP-9 expression
Source: Front Oncol. 2025 Feb 17;15:1441384. doi: 10.3389/fonc.2025.1441384 (PMC11872906; doi:10.3389/fonc.2025.1441384)

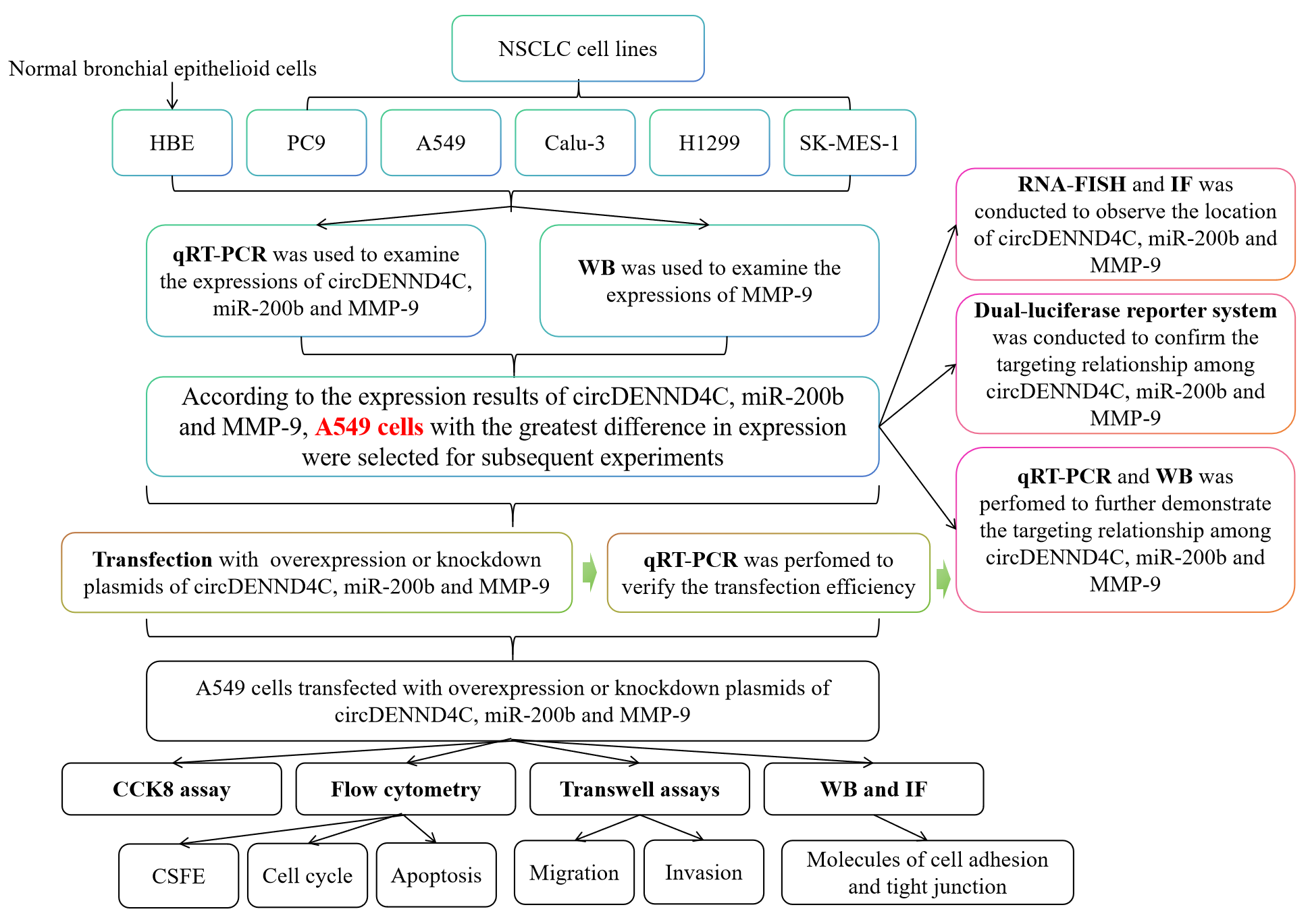

Supplement: Supplementary Figure 1 — The experimental operation flow chart of this study. The content in the blue box is the verification of the expression of circDENN4C, miR-200b and MMP-9 in NSCLC cell lines, the content in the green box is the verification of the plasmids of circDENN4C, miR-200b and MMP-9, and the content in the red box is the verification of the targeted regulatory interaction of circDENN4C, miR-200b and MMP-9, while the content in the black box is the verification of the biological function of circDENN4C, miR-200b and MMP-9. Black bold font is the methodology, while red bold font is the cell type selected for the targeted relationship validation and biological function validation experiments. [file Image1.tif]

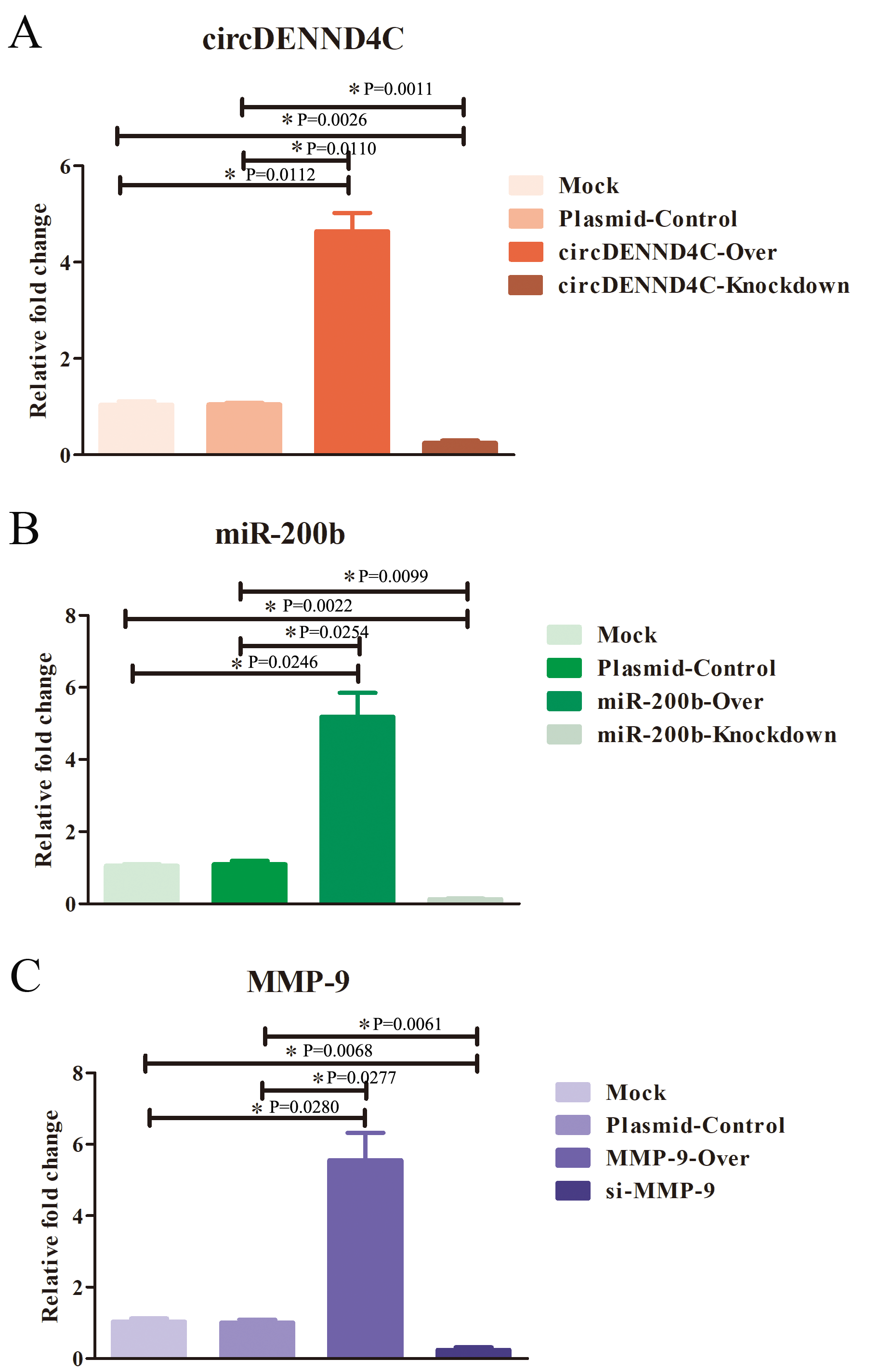

Supplement: Supplementary Figure 2 — Validation of plasmid transfection efficiency. qRT-PCR determined the levels of circDENND4C (A), miR-200b (B) and MMP-9 (C) in A549 cells after the transfection. The data are presented as the mean ± SEM. *p < 0.05 and the detailed p-values have been labeled in the graph. [file Image2.tif]
